# Supplementary material for: Synthesis of Enantiopure (S)-Atenolol by Utilization of Lipase-Catalyzed Kinetic Resolution of a Key Intermediate
Source: Int J Mol Sci. 2024 Mar 20;25(6):3497. doi: 10.3390/ijms25063497 (PMC10970586; doi:10.3390/ijms25063497)
Supplement: Supplementary file 1 [file ijms-25-03497-s001.zip › ijms-2900705-supplementary.pdf]

Suppelementary Materials

Synthesis of Enantiopure (*S*)-Atenolol by Utilization of Lipase-Catalyzed Kinetic Resolution of a Key Intermediate

Mari Bergan Hansen,[1] Anna Lifen Tennfjord,[1] Fredrik Heen Blindheim,[1] Lucas Hugo Yvan Bocquin[1] and Elisabeth Egholm Jacobsen[1]*

[1] Department of Chemistry, Norwegian University of Science and Technology (NTNU), Høgskoleringen 5 , N-7491 Trondheim , Norway.

HPLC chromatograms

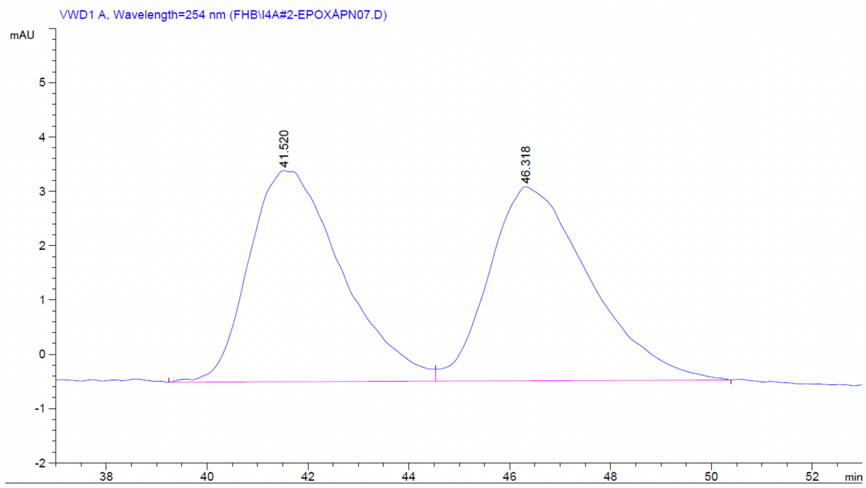

Figure S1. 2-(4-(3-Chloro-2-hydroxypropoxy)phenyl)acetamide (**2a**), resolved on a Chiralcel OD-H column (250 mm L x 4.6 mm ID, 5 µm particle size), isocratic eluent *i*-PrOH: *n*-hexane:, 17:83, 1.0 mL/min. t_R (*S*)-**2a** = 41.52 min, t_R (*R*)-**2a** = 46.31 min, R_S = 1.74.

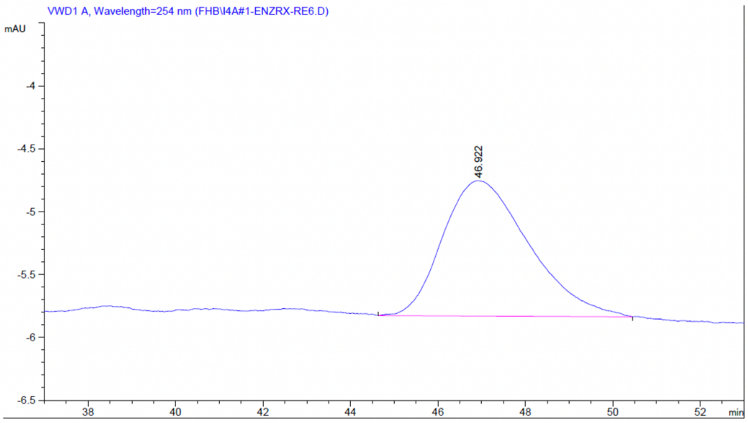

Figure S2. (*R*)-2-(4-(3-chloro-2-hydroxypropoxy)phenyl)acetamide ((*R*)-**2a**), analyzed on Chiralcel OD-H column (250 mm L x 4.6 mm ID, 5 µm particle size), isocratic eluent *i*-PrOH: *n*-hexane:, 17:83, 1.0 mL/min, t_R (*R*)-**2a** = 46.92 min, > 99% *ee*.

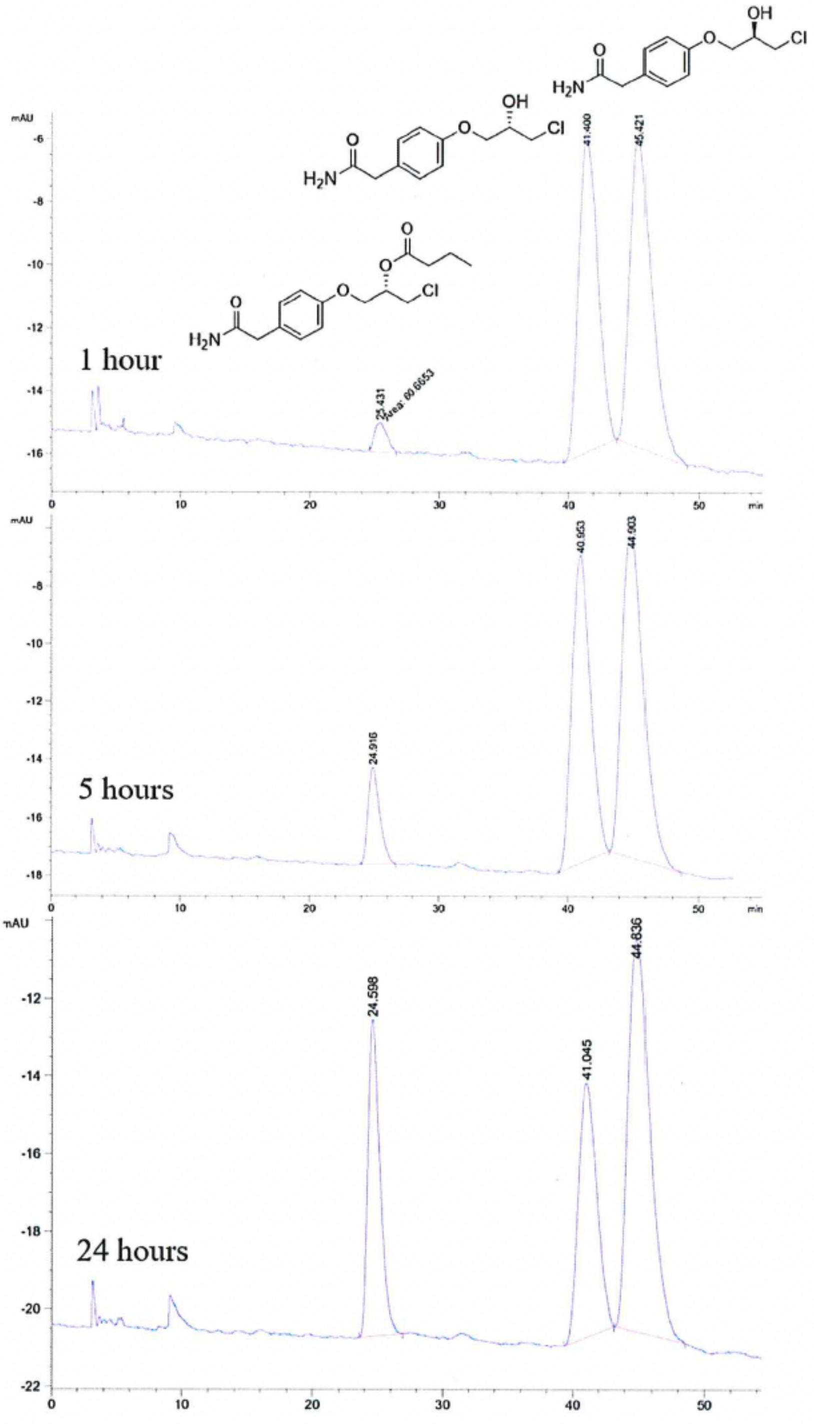

Figure S3: Kinetic resolution of **2a** catalyzed by CALB after 1, 5 and 24 hours of reaction. HPLC analyses performed on a Chiralcel OD-H column (250 mm L x 4.6 mm i.d, 5 μm particle size) with isocratic eluent *i*-PrOH:*n*-hexane, 17:83, flow 1 mL/min. This gave unseparated ester enantiomers t_R (*R*/*S*)-**2b** = 25.37 min, t_R (*S*)-**2a** = 41.66 min and t_R (*R*)-**2a** = 45.22 min with R_S = 1.4.

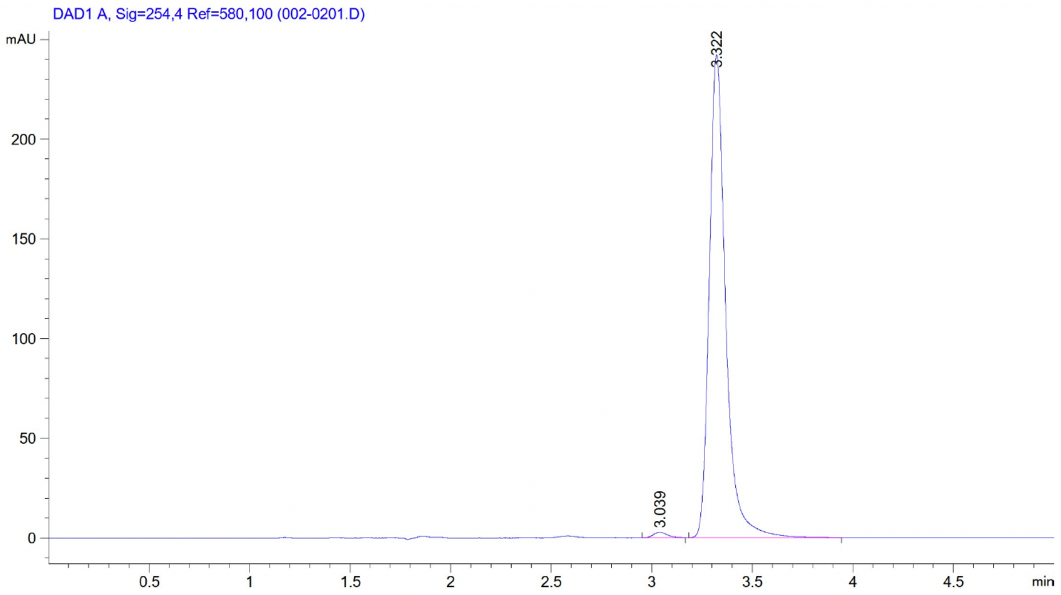

Figure S4: HPLC-chromatogram of chlorohydrin **2a** (t_R = 3.32 min) analyzed on an (achiral) Agilent Zorbax Eclipse XBD-C18 column (150 mm L × 4.6 mm i.d., 5 µm particle size) with an isocratic mobile phase H_2O:MeCN,25:75 over 5 min, flow 1.0 mL/min.
